# How does obesity affect mortality through blood pressure and blood glucose in Chinese and US citizens? Insights from a causal mediation analysis of two large cohorts

**DOI:** 10.7189/jogh.13.04032

**Published:** 2023-03-24

**Authors:** Qi Huang, Xiantong Zou, Pei Gao, Xueyao Han, Xianghai Zhou, Linong Ji

**Affiliations:** 1 Department of Endocrinology and Metabolism, Peking University People’s Hospital, Beijing, China; 2 Department of Epidemiology and Biostatistics, School of Public Health, Peking University, Beijing, China

## Abstract

**Background:**

Obesity, which has reached the scale of a global pandemic, is a leading cause of premature death. It is unclear to what extent its effect on mortality was driven by blood pressure or glucose levels in people of different ethnicities.

**Methods:**

We conducted a causal mediation analysis to estimate the mediation effect of blood pressure and glucose between body mass index (BMI) or waist-hip ratio (WHR) on mortality based on data from the China Kadoorie Biobank (CKB) (n = 458 385) and US National Health and Nutrition Examination Survey (NHANES) (1999-2008, n = 20 726).

**Results:**

The WHR's effect on mortality was mediated by blood pressure and glucose in the CKB data set by 38.7% (95% confidence interval (CI) = 34.1, 43.2) and 36.4% (95% CI = 31.6, 42.8), whereas in NHANES by 6.0% (95% CI = 2.3, 8.3) and 11.2% (95% CI = 4.7, 22.7), respectively. For associations between BMI and mortality in subjects with overweight or obesity, the mediator proportion of blood pressure and glucose was 49.4% (95% CI = 40.1, 62.5) and 16.9% (95% CI = 13.6, 22.9) in CKB and 9.10% (95% CI = 2.2, 25.9) and 16.7% (95% CI = 7.3, 49.0) in NHANES, respectively. We stratified the patients by their blood glucose, blood pressure level, or both into four groups. The effect of WHR on mortality was comparable across subgroups in either cohort. The associations between BMI and mortality were stronger in patients with higher blood pressure in CKB (*P* = 0.011) and blood glucose in NHANES (*P* = 0.035) in patients with overweight and obesity.

**Conclusions:**

The relationship between WHR and mortality in the CKB data set was potentially caused by blood pressure and glucose to a much greater extent than in the NHANES one. The effect of BMI influenced by blood pressure was significantly higher among Chinese individuals with overweight and obesity. These results implicate a different intervention strategy is required for blood pressure and blood glucose in China and US to prevent obesity and obesity-related premature death.

The prevalence of obesity has risen considerably in many countries since the 1980s [1]. As a global health issue, obesity predisposes people to diabetes, hypertension, stroke, coronary heart disease, and premature death [2]. Obesity-induced metabolic dysfunction activates the neurohormonal system and inflammatory cytokines, causing rises in cardiovascular diseases (CVD) and mortality [3,4]. The impact of excess weight on blood pressure and cholesterol accounts for nearly half of the increased risk of CVD [5], and efforts on controlling metabolic profiles, including blood glucose, blood pressure, and blood lipids, weaken the effect of obesity on related outcomes [6]. However, the prevalence of obesity along with substantial metabolic disorders and cardiovascular diseases remains high in emerging nations, particularly China, due to disparity in health resources, lifestyle, and physiology [7]. Concerns have been raised over whether control strategies should be designed to be country-specific, highlighting the need to understand the unique role of blood glucose or blood pressure in mediating the effect of obesity on mortality or cardiovascular mortality across regions or ethnicities.

The body mass index (BMI) is a reliable proxy for general obesity in describing the status of excess body fat, while waist circumference (WC) or waist-to-hip ratio (WHR) are utilized to evaluate central obesity. In previous studies, the relationship between BMI and all-cause mortality was U-shaped and the point of lowest risk varied across cohorts, depending on their ethnicity, smoking behavior, and comorbidities [2,8,9]. Neverthless, the relationship between mortality was J-shaped with WC and monotone with WHR [10]. WHR seemed to have a more direct and explainable association with mortality than WC [11], but more evidence of multi-cohort studies are needed. In this study, we used BMI and WHR were as indicators of obesity and central obesity.

Previous studies primarily employed Cox regression to assess the percentage of excess risk in CVD events mediated by metabolic profiles [12,13]. In recent years, causal mediation analysis has increasingly been used to investigate the causal interpretation of a parameter on the association between exposure and outcome, and explore potential causal relationship [14-16]. We conducted causal mediation analysis in two large epidemiological cohorts from China and the United States to investigate causal pathway between general obesity or central obesity and all-cause and cardiovascular mortality through blood pressure and glucose, and whether this effect varied across different ethnicities.

## METHODS

### Study population

The China Kadoorie Biobank (CKB) is an open-ended prospective cohort study of 512 726 adults aged 35-74 from five urban and five rural regions in China, conducted between 2004 and 2008. Detailed designs and methods have been documented previously [17,18]. We excluded underweight participants (BMI≤18.5 kg/m^2^, n = 21 419) or those with an extreme low level of waist-hip-ratio (WHR≤0.5, n = 17). To avoid reverse causation, we excluded people with a history of cardiovascular diseases (including chronic heart disease, stroke, or transient ischaemic attack) or cancers (n = 26 348), and those who were lost to follow up in the first three years (n = 6557). The final analysis included 458 385 participants.

The US National Health and Nutrition Examination Survey (NHANES) is a nationally representative study with a national, multistage, stratified, clustered probability sampling design [19] which was previously described elsewhere [20]. To match the follow-up period of CKB, we considered 27 172 adults from NHANES cycle 1999-2008. We excluded participants with missing data for BMI (n = 1683), underweight (BMI≤18.5 kg/m^2^, n = 512), with an extremely low level of waist-hip-ratio (WHR≤0.5, n = 0), those previously diagnosed as cardiovascular diseases or cancers (n = 3964), and censored in the first three years (n = 376). We included 20 726 participants in the final analysis (Figure S1 in the **Online Supplementary Document**).

### Definition of exposure

We defined general obesity with BMI, which was calculated as weight in kilograms divided by the square of standing height in meters. We defined central obesity with waist circumference (WC) or WHR, measuring WC at the midpoint between the lowest rib and iliac crest and calculating WHR as WC divided by hip circumference (the maximum circumference around the backside). Due to lack of information on hip circumference in NHANES 1999-2008, we estimated WHR using a linear regression model from other NHANES cycles (**Online Supplementary Document**). We defined overweight as a BMI of 24-27.9 kg/m^2^ in China and 25-29.9 kg/m^2^ in US, while we defined obesity as a BMI above 28 kg/m^2^ in China and above 30 kg/m^2^ in US [7]. We measured central obesity as a WHR above 0.85 in females and above 0.9 in males in both countries.

### Measurements of metabolic mediators

Blood pressure and blood glucose were key metabolic mediators for our investigation because blood lipid profiles were not accessible in CKB. We assessed the mediators and the measurement of obesity simultaneously in both cohorts (**Online Supplementary Document****)**. We identified hypertension when a respondent reported at baseline or had elevated blood pressure at examination (systolic blood pressure (SBP)≥140 mm Hg or diastolic blood pressure (DBP)≥90 mm Hg). We identified diabetes if a participant had previously been diagnosed with diabetes or found elevated blood glucose levels (random plasma glucose (RPG)≥11.1 mmol/L or fasting plasma glucose (FPG)≥7.0 mmol/L in CKB; glycated hemoglobin (HbA_1c_)≥6.5% or FPG≥7.0 mmol/L in NHANES). For primary analysis, we chose mean SBP to represent blood pressure for the primary analysis, and RPG (in CKB) or HbA_1c_ (in NHANES) to represent blood glucose. We tested other accessible metabolic mediators in NHANES in an exploratory analysis, including total cholesterol, triglyceride, low-density lipoprotein cholesterol, high-density lipoprotein cholesterol, C-reactive protein, uric acid, blood nitrogen, serum creatinine, homocysteine, vitamin B12, total protein, albumin, alanine transaminase, aspartate aminotransferase, white blood count, red blood count, hemoglobin, and Vitamin D.

### Assessment of covariates

Information on demographic and socio-economic factors (including age, sex, region (only in CKB), race (only in NHANES), occupation, education levels, household income), smoking status, alcohol consumption, medical history was collected using interviewer-administered questionnaires. Smoking status was classified as ever/never smoking and current smoking and alcohol consumption as non-drinking and ever drinking (including ex-regular drinking and current drinking in CKB; drinking at least one alcoholic drink each day for females or drinking at least two alcoholic drinks each day for males in NHANES). Household income levels were classified as very low (less than ¥10 000 per year in CKB/less than US$10 000 in NHANES), low (¥10 000-19 999/US$10 000-24 999), medium (¥20 000-34 999/US$25 000-54 999) and high (more than ¥35 000/more than US$55 000) level.

### Measurement of outcomes

In CKB, participants were followed up till death, loss to follow up or December 31, 2015. Mortality was obtained through China’s Center for Disease Control (CDC) Disease Surveillance Points system, with annual confirmation through local residential and administrative records. Deaths were classified by the International Classification of Diseases 10 (ICD-10). The primary outcome was death from any causes, while the secondary outcome was death from cardiovascular disease excluding non-fatal hypertension (ICD-10: I00-I09, I16-I25, I27-I88). In NHANES, mortality data was acquired through linkage to the National Death Index to 31 December 2015. Because of the limited sample power, we examined only the primary outcome in NHANES.

### Statistical analysis

In analyses related to NHANES, we accounted for sample weights, stratification, and clustering to make an unbiased variance due to its complex, multistage, probability cluster survey design. We described baseline characteristics in both cohorts as mean (standard deviation (SD)) for continuous variables and count (percentage) for categorical variables.

**Figure 1:**
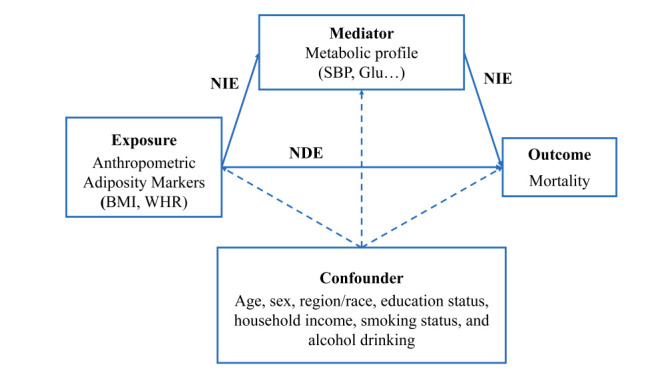
Direct acyclic graph for mediation analysis. The diagram describes the relationship between obesity (exposure) and mortality (outcome) mediated by blood pressure and glucose (mediators). The exposure-mediator effects were estimated by a linear model, and the exposure-outcome and mediator-outcome effects were estimated by accelerated failure time model, both adjusted by confounders. NDE – natural direct effects, NIE – natural indirect effects.

We used a Imai’s counterfactual framework for causal mediation analysis [21,22]. Associations between exposure (adiposity) on the outcome (mortality) would be decomposed into indirect associations mediated by the mediators (natural indirect effect (NIE)) and associations not mediated by mediator (natural direct effect (NDE)) (**Figure 1**). We therefore estimated the proportion of the association mediated by mediators (NIE/(NDE + NIE) or NIE/TE). We used SBP, RPG (in CKB), and HbA_1c_ (in NHANES) as main mediators in the main analysis. For exploratory analysis, we log-transformed other potential mediators in advance if distributions were abnormal. We imputed missing data by multiple imputation method only in main analysis, as the reliability of multiple imputations was limited in the exploratory analysis due to missing data. We applied a linear model estimating the effect of exposure on mediators and a Weibull accelerated failure time (AFT) model estimating effects of exposure and mediator on outcome, adjusting both for age, sex, region (CKB only), race (NHANES only), education status, household income, smoking status, and alcohol drinking, which were common confounders between adiposity-mortality relationships in epidemiological studies [23,24]. We estimated the standard error (SE) of parameters in the models via quasi-Bayesian Monte Carlo simulation with 100 replications. We evaluated the performance of framework by the square root of the differences between predicted values and observed values (root mean square deviation (RMSE)) for linear models and c-index for AFT models, with 10-fold cross-validation. To account for the combined effect of multiple mediators, we tried to estimate path-specific effects along multiple pathways [25,26].

We also estimated the role of blood pressure and glucose at segment levels. Using Cox models, we stratified associations between adiposity and mortality by quartiles of SBP, quartiles of blood glucose. We calculated the *P* for interaction (*P*_interaction_) from a likelihood ratio test by testing interaction term between linear risk groups and adiposity index. We also estimated associations in subgroups with low pressure and glucose, high pressure and low glucose, low pressure and high glucose, both high pressure and glucose, by median of SBP and glucose.

For sensitivity analyses, we repeated our main analysis with substituted mediators, such as hypertension status, diabetes status, and fasting glucose. We also repated mediation analyses in a different age (cut-off value at 65), sex, and racial/regional subgroups to investigate potential heterogeneous effects. To avoid falsely positive results risk by having different cut-off values in two data sets, we repeated the analysis with the BMI cut-off of 25 kg/m^2^ in CKB.

We performed all analyses using R version 4.2.0 (R Foundation for Statistical Computing, Vienna, Austria) and its “Mediation” package for the main mediation analysis. We set the statistical significance at *P* < 0.05 (two-sided).

## RESULTS

The baseline characteristics of participants in the two cohorts are presented in Table S1 in the **Online Supplementary Document**. During an average of 9.14 years of follow-up, 22 422 deaths and 10 726 cardiovascular deaths were recorded in CKB. During an average of 11.4 years of NHANES follow-up, 1863 deaths were documented.

### Mediation role of blood pressure and blood glucose

In CKB, the percentages of correlations between WHR and all-cause mortality mediated by SBP and RPG were 38.7% (95% confidence interval (CI) = 34.1, 43.2) and 36.6%, respectively (95% CI = 31.6, 42.8). In NHANES, 6.0% (95% CI = 2.3, 8.3) of WHR-mortality association was mediated by SBP, while 11.2% (95% CI = 4.7, 22.7) was mediated by HbA_1c_. We conducted the mediator analysis of BMI separately for persons with BMI<25 and ≥25 kg/m^2^. In individuals of normal weight, per SD increase in BMI was associated with longer survival time. In individuals with overweight and obesity, blood pressure and glucose caused 49.4% (95% CI = 40.1, 62.5) and 16.9% (95% CI = 13.6, 22.9) of the total effect of BMI on all-cause mortality in CKB and 9.1% (95% CI = 2.2, 25.9) and 16.7% (95% CI = 7.3, 49.0) in NHANES (**Table 1** and **Table 2**). The mediation models demonstrated high and identical performance in both cohorts (Table S2-S3 in the **Online Supplementary Document**).

**Table 1:** Mediation analysis of delayed years of all-cause mortality for per SD increase of WHR/BMI in CKB*

	WHR		BMI<24 kg/m^2^			BMI≥24 kg/m^2^			
	Estimation (95% CI)	Mediation proportion, % (95% CI)	Estimation (95% CI)	Mediation proportion, % (95% CI)		Estimation (95% CI)		Mediation proportion, % (95% CI)	
**Model 1: Mediated by SBP**								
TE	-1.7 (-1.5, -2.0)	38.7 (34.1, 43.2)	1.2 (0.9, 1.4)	-42.3 (-58.8, -33.6)	-1.4 (-1.8, -1.1)		49.4 (40.1, 62.5)	
NDE	-1.1(-1.3, -0.9)		1.7 (1.4, 1.9)		-0.7 (-1.1, -0.4)			
NIE	-0.7 (-0.7, -0.6)		-0.5 (-0.5, -0.5)		-0.7 (-0.8, -0.7)			
**Model 2: Mediated by RPG**								
TE	-1.4 (-1.6, -1.2)	36.4 (31.6, 42.8)	1.2 (0.9, 1.4)	-9.1 (-13.1, -7.3)	-1.2 (-1.6, -0.9)		16.9 (13.6, 22.9)	
NDE	-0.9 (-1.1, -0.7)		1.3 (1.0, 1.5)		-1.0 (-1.2, -0.7)			
NIE	-0.5 (-0.5, -0.5)		-0.1 (-0.1, -0.1)		-0.2 (-0.2, -0.2)			

SD – standard deviation, WHR – waist-hip ratio, BMI – body mass index, CI – confidence interval, TE – total effect, NDE – natural direct effect, NIE – naturel indirect effect, SBP – systolic blood pressure, RPG – random plasma glucose

*We used linear model to estimate exposure-mediator effect and accelerated failure time model to estimate exposure-outcome and mediator-outcome effects. The total effect represented the association between WHR/BMI on all-cause mortality. Natural indirect effect represented the association mediated by the mediators (blood pressure or glucose). Natural direct effect represented the association not mediated by selected mediators. We calculated the mediation proportion by NIE/TE and estimated effects as delay years of all-cause death. We estimated 95% Cis by quasi-Bayesian Monte Carlo simulation with 100 replications. We adjusted all analyses for age, sex, region, education status, household income, current smoking, and alcohol drinking.

**Table 2:** Mediation analysis of delay years of all-cause mortality for per SD increase of WHR/BMI in US NHANES*

	WHR		BMI<25 kg/m^2^			BMI≥25 kg/m^2^			
	Estimation (95% CI)	Mediation proportion, % (95% CI)	Estimation (95% CI)	Mediation proportion, % (95% CI)		Estimation (95% CI)		Mediation proportion, % (95% CI)	
**Model 1: Mediated by SBP**								
TE	-6.0 (-8.4, -3.5)	6.0 (2.3, 8.3)	7.7 (4.0, 12.0)	-2.7 (-4.9, -0.8)	-4.0 (-6.4, -1.3)		9.10 (2.2, 25.9)	
NDE	-5.7 (-8.2, -3.2)		7.8 (4.1, 12.0)		-3.7 (-6.1, -1.0)			
NIE	-0.4 (-0.5, -0.2)		-0.2 (-0.4, -0.1)		-0.4 (-0.8, -0.1)			
**Model 2: Mediated by HbA_1c_**								
TE	-5.7 (-8.1, -3.2)	11.2 (4.7, 22.7)	7.6 (4.4, 11.9)	-0.9 (-3.2, 0.2)	-4.0 (-6.1, -1.3)		16.7 (7.3, 49.0)	
NDE	-5.1 (-7.5, -2.7)		7.7 (4.5, 12.0)		-3.3 (-5.5, -0.8)			
NIE	-0.7 (-1.1, -0.3)		-0.1 (-0.2, 0.01)		-0.6 (-1.0, -0.4)			

SD – standard deviation, WHR – waist-hip ratio, BMI – body mass index, CI – confidence interval, TE – total effect, NDE – natural direct effect, NIE – naturel indirect effect, SBP – systolic blood pressure, HbA_1c_ – glycated hemoglobin.

*We used a linear model for estimate exposure-mediator effect and an accelerated failure time model to estimate exposure-outcome and mediator-outcome effects. The total effect represented the association between WHR/BMI on all-cause mortality. Natural indirect effect represented the association mediated by the mediators (blood pressure or glucose). Natural direct effect represented the association not mediated by selected mediators. We calculated mediation proportion by NIE/TE and estimated effects by delay years for all-cause mortality. We estimated 95% CIs by quasi-Bayesian Monte Carlo simulation with 100 replications. We adjusted all analyses for age, sex, race, education status, household income, current smoking, and alcohol drinking

Blood pressure and glucose mediated similar proportions between WHR/BMI and cardiovascular mortality (Table S4 in the **Online Supplementary Document**) in CKB. After considering combined effect of metabolic factors, direct effect of WHR on mortality was not significant (mediation proportion = 12.5%; 95% CI = -2.3, 27.3) in CKB, while it remained substantial in NHANES (79.7%; 95% CI = 61.8, 97.6) (Table S5-7 in the **Online Supplementary Document**).

### Association between obesity and mortality in subgroups stratified by blood pressure and glucose

**Figure 2:**
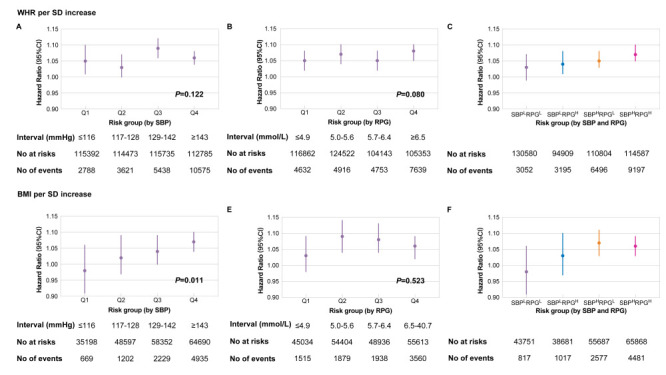
Risk for all-cause mortality per SD increase in WHR/BMI stratified by blood glucose and pressure in CKB. **Panel A.** Hazard ratio (95% CI) for all-cause mortality per SD increase in WHR stratified by quartiles of SBP. **Panel B.** Hazard ratio (95% CI) for all-cause mortality per SD increase in WHR stratified by quartiles of RPG. **Panel C.** Hazard ratio (95% CI) for all-cause mortality per SD increase in WHR stratified by a combination of SBP and RPG. **Panel D.** Hazard ratio (95% CI) for all-cause mortality per SD increase in BMI stratified by quartiles of SBP. **Panel E.** Hazard ratio (95% CI) for all-cause mortality per SD increase in BMI stratified by quartiles of RPG. **Panel F.** Hazard ratio (95% CI) for all-cause mortality per SD increase in BMI stratified by a combination of SBP and RPG. Hazard ratios were assessed by by Cox regression models adjusted for age, sex, region, education status, household income, current smoking, and alcohol drinking. *P*-values were estimated by comparing models adding an interaction term between risk group (as linear) and WHR/BMI against the original models, using the likelihood ratio test. Glucose or blood pressure intervals were defined as quartiles. Participants were divided into four groups using the median of SBP and RPG. SBP – systolic blood pressure, RPG – random plasma glucose, SBPLRPGL – SBP lower than the median and RPG lower than the median, SBPHRPGL – SBP higher than the median and RPG lower than the median, SBPLRPGH – SBP lower than the median and RPG higher than the median, SBPHRPGH – SBP higher than the median and RPG higher than the median.

In CKB, there was no difference in associations between WHR and all-cause mortality among quartiles of SBP or RPG (**Figure 2****,** panel A-B). WHR elevated the mortality risk in subgroup with both high SBP and RPG but not in subgroups with either low SBP or RPG, or both (**Figure 2****,** panel C). In patients with obesity and overweight, the HRs of BMI on mortality increased with increasing SBP quartiles (*P* = 0.011, **Figure 2****,** panel D). No significant trends were found in different levels of glucose (**Figure 2****,** panel E). There was a significant positive association between BMI and mortality in subgroups with higher SBP, regardless of the RPG levels (**Figure 2****,** panel F).

**Figure 3:**
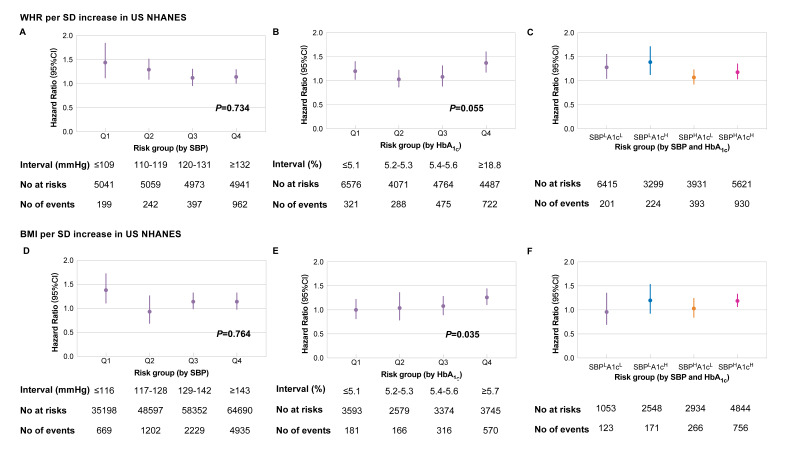
Risk for all-cause mortality per SD increase in WHR/BMI stratified by blood glucose and pressure in US NHANES. **Panel A.** Hazard ratio (95% CI) for all-cause mortality per SD increase in WHR stratified by quartiles of SBP. **Panel B.** Hazard ratio (95% CI) for all-cause mortality per SD increase in WHR stratified by quartiles of HbA1c. **Panel C.** Hazard ratio (95% CI) for all-cause mortality per SD increase in WHR stratified by a combination of SBP and HbA1c. **Panel D.** Hazard ratio (95% CI) for all-cause mortality per SD increase in BMI stratified by quartiles of SBP. **Panel E.** Hazard ratio (95% CI) for all-cause mortality per SD increase in BMI stratified by quartiles of HbA1c. **Panel F.** Hazard ratio (95% CI) for all-cause mortality per SD increase in BMI stratified by a combination of SBP and HbA1c. Hazard ratios were assessed by Cox regression models adjusted for age, sex, race, education status, household income, current smoking, and alcohol drinking. *P*-value was estimated by comparing models adding an interaction term between risk group (as linear) and WHR/BMI against the original models, using the likelihood ratio test. Glucose or blood pressure intervals were defined as quartiles. Participants were divided into four groups using the median of SBP and RPG. SBP – systolic blood pressure, RPG – random plasma glucose, HbA1c (A1c) – glycated hemoglobin. SBPLA1cL – SBP lower than median and HbA1c lower than median, SBPHA1cL – SBP higher than median and HbA1c lower than median, SBPLA1cH – SBP lower than median and HbA1c higher than median, SBPHA1cH – SBP higher than median and HbA1c higher than median.

In the NHANES, the associations between WHR and mortality were comparable across subgroups stratefied by SBP (*P* = 0.734, **Figure 3****,** panel A) and HbA_1c_ (*P* = 0.055, **Figure 3****,** panel b) levels. WHR was significantly associated with mortality except in the subgroup with high SBP and low HbA_1c_ (**Figure 3****,** panel C). In patients with overweight and obesity, the hazard ratios (HRs) of BMI on mortality increased along with HbA_1c_ (*P* = 0.035), but not SBP (*P* = 0.764) (**Figure 3****,** panel D-E). HRs of BMI on mortality were only higher in subgroups with greater HbA_1c_ and SBP (**Figure 3****,** panel F).

### Sensitivity analysis

Substituting mediators to hypertension status, diabetes status, and fasting glucose did not affect the results of the primary analysis (Table S8-9 in the **Online Supplementary Document**). The change of BMI cut-off also remained constant in the primary analysis (Table S10 in the **Online Supplementary Document**). Subgroup analysis suggested the mediation role of SBP between WHR and mortality may be higher in younger people (mediator effect of SBP in people <65 vs ≥65 years: 42.8% (95%CI = 36.6, 49.3) vs 24.7% (95% CI = 19.7, 32.0). However, the mediation proportions were highly consistent in other subgroups (Table S11-12 in the **Online Supplementary Document**).

## DISCUSSION

The association between WHR and mortality was attributable to blood pressure and glucose on a rather larger scale in the Chinise cohort (CKB) than the US cohort (NHANES). Similar proportions of the association between BMI and mortality in individuals with overweight or obesity were mediated by blood glucose in both cohorts, while blood pressure mediated double the proportion of blood glucose in CKB and one-half the proportion of blood glucose in NHANES.

A previous cross-sectional study found a higher risk of hypertension and diabetes in the Chinese males compared to the White males with the same BMI [27], but failed to assess the potential outcomes. A pooled analysis from 97 prospective cohort studies [12] estimated the hazard ratio of the effects of obesity on coronary heart disease and stroke caused by metabolic mediators, but did not establish a causal relationship between obesity and mortality. Our counterfactual framework showed potential roles of metabolic factors mediating the disparity of obesity and central obesity-related mortality between Chinese and US. To achieve the best fit of the model and increase the capability of establishing causal relationships, we controlled exposure-outcome, mediator-outcome, and exposure-mediator confounding, and accounted for exposure-induced mediator-outcome confounding. We discovered that blood glucose and blood pressure accounted for a much greater proportion of the WHR-related and BMI-related mortality in China than in the US.

The disparity in the triangle relationship of obesity, metaoblic profile, and mortality between Chinese and US is supported by evidence from other studies. Health metabolic profiles in obesity, also called metabolically healthy obesity (MHO) was associated with a lower risk of major vascular events in Chinese (HR = 1.08,95% CI = 1.02, 1.14) [28] than in US (HR = 1.39, 95% CI = 1.15, 1.68) [29], supporting a greater mediation role of metabolic factors in Chinese population. Sbesity-related premature deaths can be largely explained by blood pressure and blood glucose in China, but more potential mechanisms should be investigated in US. The exploratory analysis of the possible relevant mediators found that serum albumin and vitamin D might mediate the effect of excess adiposity on mortality (Table S13 in the **Online Supplementary Document**), indicating the importance of nutritional factors in US citizens.

Our mediation analysis identified the differential effect of blood glucose and blood pressure on obesity/central obeisty related mortality in both cohorts, further supported by our stratification analysis. The association between WHR and mortality was attenuated in individuals with the presence of both low blood glucose and blood pressure in Chinese. We found blood pressure alone mediated more than half the effect of BMI on mortality in CKB; increasing blood pressure was associated with a linear increase in BMI-related mortality, and the association between BMI and mortality was prominent in both high blood pressure regardless of blood glucose level. However, in NHANES, glucose played a more significant mediation role, and WHR and BMI were both significantly correlated with mortality in the highest quartile of blood glucose.

Our data provided insights to metabolic control strategies in both countries in this global pandemic of obesity. Controlling blood pressure in China was shown to be of utmost importance to reduce obesity-related premature death in the country. In fact, less than a half of Chinese with hypertension were treated regardless of BMI levels [30], while the rate of antihypertensive medication use were greater than 70% [31]. The spectrum of causes of death differed between China and the US. East Asians had a high incidence of stroke, with high blood pressure being the main risk factor [30]. A pooled analysis suggested that the regional heterogeneity in stroke risk appeared to disappear after adjusting for blood pressure, indicating that hypertension played a central role [9]. Also, blood glucose control was more prominent than blood pressure control in the US. This was supported by the subgroup analysis from the University of Illinois Cohort (UIC) of Patients, Family, and Friends, which suggested each unit increase in genetic risk scores was only associated with obesity in patients with diabetes (odds ratio (OR) = 2.24; 95% CI = 1.36, 3.70) and not hypertension (OR = 1.43; 95% CI = 0.93, 2.18) [32]. However, the larger proportion of mediator effect of blood pressure may be a “low-hanging fruit” effect, because CKB is more intervention-naive and even smaller effects are immediately transferred to better mortality profiles. In contrast, the US population had already depleted this primordial effect and remained to have different mortality trajectories. The difference of intervention status on blood pressure and blood pressure on the mediation effect in two cohorts could be further assessed if data were available.

To address the disparity of anthropological characters other than ethnicity between NHANES and CKB (Table S11-12 in the **Online Supplementary Document**), we ran sensitivity analyses to support our findings. We stratified the participants according to their age, gender, and region and the effect size of blood pressure and blood glucose on mortality recapitulated the whole group in both cohorts. For each subgroup, we observed rather larger effects in CKB than in NHANES. In both cohorts, the mediator effect of the blood pressure and blood glucose was higher in younger participants. It is possible that the overall effects of the mediators were higher as CKB enrolled participants from 18 years rather than 30 years. We also found the mediation effect was attenuated in Asian American (possibly 2/3 of the other race in NHANES), although this was possibly due to a limited subgroup sample size. This indicates that the different results between NHANES and CKB were attributable to regional difference rather than ethical difference.

To our knowledge, this is the first and largest study to explore the potential causal effect of obesity on mortality based on metabolic profiles using mediation analysis methods. However, it has some limitations. The design and methodology of NHANES differed from that of CKB, which limited our ability to compare the two populations. We used random blood glucose in CKB instead of HbA_1c_ as the proxy for blood glucose, possibly introducing bias in estimation. Also, CKB was not designed to represent the general Chinese population, limiting the external validity of our findings. Likewise, we estimated WHR in NHANES by a regression model instead of measurements, reducing the credibility of the results. However, the predictive ability of the equations evaluated by other cycles of NHANES meant that our results were highly accurate. Furthermore, the indices for obesity might change over time, but follow-up information was limited, especially in NHANES. Due to insufficient data on other metabolic profiles in CKB, we were not able to estimate their mediated role in mortality risk. We were also unable to estimate the significant association between obesity and cardiovascular mortality due to the limited sample size in NHANES. Additionally, we observed a lower self-correlation coefficient between WHR at baseline and re-survey, which means that the real association between WHR and mortality may be underestimated in NHANES. Insufficient repeatability may also hinder the application of WHR. Finally, though we adjusted for many socioeconomic variables and performed a series of sensitivity analyses, residual confounding could have still existed, weakening our causal interpretation.

## CONCLUSIONS

From an epidemiological perspective, blood pressure and blood glucose play larger mediation roles between obesity/central obesity and mortality in China than in US, which may result from differences in region, anthropology and methodology of data collection of the two cohorts. Additionally, we found that different strategies for blood pressure and blood glucose control should be implemented in China than in the US to reduce premature mortality associated with obesity and central obesity.

## Additional material


Online Supplementary Document
