# Supplementary material for: How does obesity affect mortality through blood pressure and blood glucose in Chinese and US citizens? Insights from a causal mediation analysis of two large cohorts
Source: J Glob Health. 2023 Mar 24;13:04032. doi: 10.7189/jogh.13.04032 (PMC10078858; doi:10.7189/jogh.13.04032)
Supplement: Online Supplementary Document [file jogh-13-04032-s001.pdf]

Supplementary Data

Supplementary Method

Variable Collections

In CKB, standing height was measured to the nearest 0.1 cm using a stadiometer. Weight was measured to the nearest 0.1 kg using a TBF-300 Body Composition Analyzer (Tanita Inc., Tokyo, Japan). Waist circumference and hip circumstance were measured to the nearest 0.1 cm with a soft nonstretchable tape. Blood pressure was measured twice on the unclothed right upper arm using an automated A&D UA-779 digital monitor. If the difference between the first two measurements was >10mmHg, the result would be measured for the third time. The average of the measurements was used for analysis. Random blood glucose was evaluated by on-site rapid dipstick testing from samples of 10 ml non-fasting blood. If the value was 7.8-11.0 mmol/L, the participant will take a further fasting blood glucose test on the following day.

In NHANES, body measurements were recorded by a trained examiner in the mobile examination center (MEC)(1). Blood pressure was measured by mean of measurements at least 3 times in the mobile examination centre (MEC) or during home examinations using a mercury sphygmomanometer. Glycosylated hemoglobin (HbA_{1c}) was measured from whole blood samples using high-performance liquid chromatography methods. Fasting plasma glucose levels were measured in fasting blood specimens using a hexokinase enzymatic method.

Waist-to-hip Ratio Estimations in NHANES

We estimated WHR using a stepwise linear regression model developed in NHANES III and validated in NHANES 2017-2018. In these NHANES cycles, participants with complete information on age, race, height, weight, waist circumference, hip circumference, and BMI were recruited.

A total of 16,778 participants in NHANES III and 9,804 participants in NHANES 2017-2018 consisted of the derivation cohort and validation cohort respectively. Age, sex, race, height, weight, waist circumference and BMI were used as independent predictors. Age, race and weight were excluded after stepwise method. WHR were estimated by the following formula: WHR = 0.99 + 0.074*(sex= “Male”) - 0.355* height(m) + 0.991*WC(m) - 0.016*BMI (kg/m^2). The R^2 of the model was 0.9 in the derivation cohort and 0.7 in the validation cohort.

Reference

1. National Health and Nutrition Examination Survey, N, 2017. Anthropometry Procedures Manual.

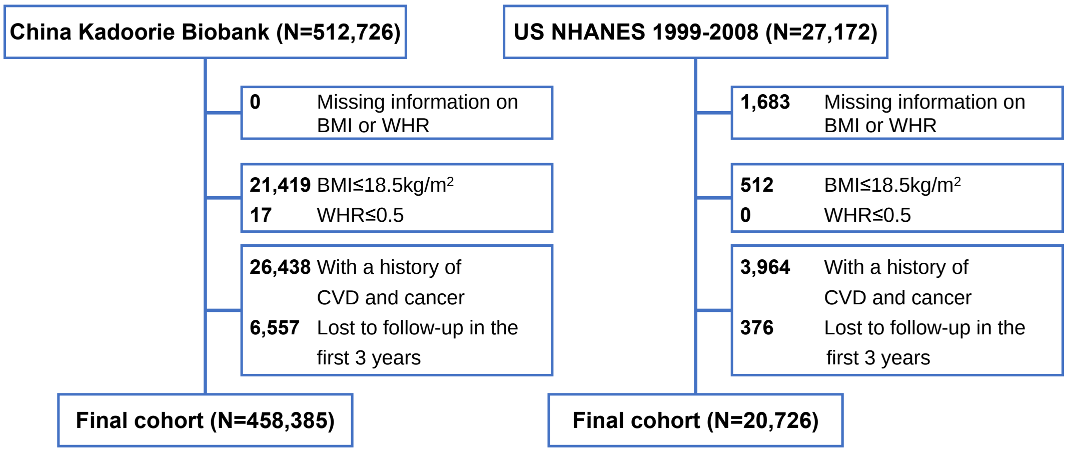

Figure S1. Study flow diagram. CVD=cardiovascular disease.

Table S1. Baseline characteristics of participants from China Kadoorie Biobank (CKB) and US National Health and Nutrition Examination Survey (US NHANES)

	CKB (n= 458,385)	US NHANES (n=20,726)
**Age (years)**	51.21 (10.38)	42.16 (15.6)
**Female (%)**	272,202 (59.4)	10,906 (51.3)
**Urban (%)**	201,180 (43.9)	--
**Race (%)**		
Hispanic	--	6,346 (14.0)
Non-Hispanic Black	--	4,522 (11.6)
Non-Hispanic White	--	9,011 (68.9)
Other Race	--	847 (5.5)
**BMI (kg/m^2)**	23.89 (3.15)	28.27 (6.3)
**Weight (kg)**	60.39 (10.28)	81.22 (20.3)
**WHR (male)**	0.91 (0.06)	0.96 (0.07)
**WHR (female)**	0.87 (0.07)	0.88 (0.07)
**WC (cm, male)**	82.6 (9.34)	99.2 (15.0)
**WC (cm, female)**	79.5 (9.04)	93.6 (15.9)
**SBP (mmHg)**	130.77 (20.9)	120.78 (16.8)
**DBP (mmHg)**	77.84 (11.02)	71.10 (12.3)
**Hypertension (%)**	150,312 (32.8)	6,429 (29.2)
**Diabetes (%)**	24,625 (5.4)	1,987 (7.3)
**Current Smoking (%)**	135,280 (29.5)	4,186 (23.5)
**≥12 years education (%)**	26,659 (5.8)	3,607 (33.3)
**Household income (%) ***		
Very low	126,621 (27.6)	1,411 (4.4)
Low	132,714 (29.0)	4,575 (16.0)
Medium	114,464 (25.0)	6,607 (31.1)
High	84,586 (18.5)	6,589 (43.2)
Unknown	--	1544 (5.2)
**Overweight (%)**	157,777 (34.4)	5618 (34.0)
**Obese (%)**	49,060 (10.7)	5104 (31.6)
**Central obesity defined by WHR (%)**	274,018 (59.8)	15,780 (75.0)

Data were described as mean (SD) for continuous variables (all continuous variables were normal distribution) and counts (percentage) for category variables. BMI=body mass index; WHR=waist hip ratio; WC=waist circumference; SBP=systolic blood pressure; DBP=diastolic blood pressure. *In CKB, <¥10,000, ¥10,000-19,999, ¥20,000-34,999 and ≥¥35,000 of household income per year respectively represented very low, low, medium and high level. In NHANES, <$10,000, $10000-24,999, $25,000-54,999 and ≥$55,000 of household income per year respectively represented very low, low, medium and high level.

Table S2. Performance of mediation models with 10-fold cross-validation in CKB

	**WHR**		**BMI<24 kg/m^2**		**BMI≥24 kg/m^2**	
Mediators	Linear model: RMSE (95%CI)	AFT model: c-index (95%CI)	Linear model: RMSE (95%CI)	AFT model: c-index (95%CI)	Linear model: RMSE (95%CI)	AFT model: c-index (95%CI)
SBP	19.1 (19.0-19.2)	0.798 (0.794-0.801)	18.2 (18.1-18.2)	0.806 (0.803-0.809)	19.3 (19.2-19.4)	0.785 (0.779-0.791)
RPG	2.18 (2.17-2.19)	0.797 (0.795-0.799)	2.02 (1.98-2.07)	0.806 (0.803-0.810)	2.40 (2.36-2.44)	0.783 (0.777-0.788)

The mediation models consisted of linear model for estimating exposure-mediator effect and accelerated failure time (AFT) model for estimating exposure-outcome and mediator-outcome effects. Both models were adjusted for age, sex, region, education status, household income, current smoking, and alcohol drinking and validated through 10-fold cross-validation. The performance of linear model was evaluated by the square root of the differences between predicted values and observed values (root mean square deviation, RMSE). The performance of AFT model was evaluated by c-index. SBP=systolic blood pressure. RPG=random plasma glucose.

Table S3. Performance of mediation models with 10-fold cross-validation in NHANES

	WHR		BMI<25 kg/m^2		BMI≥25 kg/m^2	
Mediators	Linear model: RMSE (95%CI)	AFT model: c-index (95%CI)	Linear model: RMSE (95%CI)	AFT model: c-index (95%CI)	Linear model: RMSE (95%CI)	AFT model: c-index (95%CI)
SBP	15.3 (15.0-15.7)	0.833 (0.822-0.845)	14.3 (13.6-15.1)	0.850 (0.831-0.870)	15.6 (15.4-15.9)	0.821 (0.810-0.832)
HbA_{1c}	0.90 (0.88-0.91)	0.836 (0.828-0.844)	0.70 (0.59-0.81)	0.853 (0.831-0.875)	0.97 (0.90-1.04)	0.824 (0.813-0.835)

The mediation models consisted of linear model for estimating exposure-mediator effect and accelerated failure time (AFT) model for estimating exposure-outcome and mediator-outcome effects. Both models were adjusted for age, sex, race, education status, household income, current smoking, and alcohol drinking and validated through 10-fold cross-validation. The performance of linear model was evaluated by the square root of the differences between predicted values and observed values (root mean square deviation, RMSE). The performance of AFT model was evaluated by c-index. SBP=systolic blood pressure. HbA_{1c}=glycated hemoglobin.

Table S4. Mediation analysis of delay years of cardiovascular mortality for per SD increase of WHR/BMI in CKB

	**WHR**		**BMI<24 kg/m²**		**BMI≥24 kg/m²**	
	Estimation (95%CI)	Mediation proportion, % (95%CI)	Estimation (95%CI)	Mediation proportion, % (95%CI)	Estimation (95%CI)	Mediation proportion, % (95%CI)
**Model 1: Mediated by blood pressure (SBP)**						
TE	-4.68 (-5.17, -4.17)	36.0 (32.9, 39.2)	1.99 (1.29, 2.54)	-66.1 (-106, -50.1)	-3.41 (-4.07, -2.70)	47.8 (40.2, 58.2)
NDE	-3.03 (-3.43, -2.53)		3.29 (2.62, 3.85)		-1.78 (-2.38, -1.15)	
NIE	-1.67 (-1.79, -1.56)		-1.31 (-1.44, -1.19)		-1.63 (-1.80, -1.46)	
**Model 2: Mediated by blood glucose (RPG)**						
TE	-3.65 (-4.12, -3.18)	24.5 (22.1, 28.0)	2.02 (1.37, 2.54)	-10.2 (-16.5, -7.88)	-2.86 (-3.48, -2.20)	12.9 (10.8, 16.6)
NDE	-2.75 (-3.19, -2.30)		2.23 (1.59, 2.75)		-2.49 (-3.08, -1.85)	
NIE	-0.90 (-0.95, -0.83)		-0.21 (-0.24, -0.19)		-0.37 (-0.41, -0.33)	

Linear model was used for estimate exposure-mediator effect and accelerated failure time model was used to estimate exposure-outcome and mediator-outcome effects. The total effect represented the association between WHR/BMI on cardiovascular mortality. Natural indirect effect represented association mediated by the mediators (blood pressure or glucose). Natural direct effect represented association not mediated by mediators. Mediation proportion was calculated by NIE/TE. Effects were estimated by delay time for all-cause mortality. 95% confidence interval (95%CI) was estimated by quasi-Bayesian Monte Carlo simulation with 100 replications. All analyses were adjusted for age, sex, region, education status, household income, current smoking, and alcohol drinking. TE=Total Effect. NDE=Natural direct effect.

NIE=Naturel indirect effect. SBP=systolic blood pressure. RPG=random plasma glucose.

Table S5. Multiple mediation analysis of risk of all-cause mortality per SD increase of WHR/BMI in CKB

	**WHR**	**BMI<24 kg/m²**	**BMI≥24 kg/m²**
Effect	Mediation proportion, % (95%CI)	Mediation proportion, % (95%CI)	Mediation proportion, % (95%CI)
SBP	50.7 (42.1, 59.2)	-39.6 (-51.3, -28.0)	50.0 (37.2, 62.8)
PRG	36.8 (30.0, 43.7)	-8.43 (-11.5, -5.35)	14.1 (10.2, 17.9)
Direct Effect	12.5 (-2.25, 27.3)	148 (134, 162)	35.9 (19.9, 51.8)

Proportions of path-specific effects were estimated using g-computation algorithm, including age, sex, region, education status, household income, current smoking, and alcohol drinking as covariates. Interaction effect between both mediators was no significant in all models, thus not presented. SBP=systolic blood pressure. RPG=random plasma glucose.

Table S6. Multiple mediation analysis of risk of all-cause mortality per SD increase of WHR/BMI in US NHANES

	WHR	BMI<25 kg/m²	BMI≥25 kg/m²
Effect	Mediation proportion, % (95%CI)	Mediation proportion, % (95%CI)	Mediation proportion, % (95%CI)
SBP	8.72 (-0.08, 17.5)	-2.95 (-8.70, 2.80)	15.2 (-9.51, 39.9)
HbA_{1c}	13.0 (0.38, 25.5)	-0.83 (-3.89, 2.22)	19.6 (-7.24, 46.4)
Direct Effect	79.7 (61.8, 97.6)	104 (97.6, 110)	65.1 (18.3, 120)

Proportions of path-specific effects were estimated using g-computation algorithm, including age, sex, race, education status, household income, current smoking, and alcohol drinking as covariates. Interaction effect between both mediators was no significant in all models, thus not presented. SBP=systolic blood pressure. HbA_{1c}=glycated hemoglobin.

Table S7. Multiple mediation analysis of risk of cardiovascular mortality per SD increase of WHR/BMI in CKB

	**WHR**	**BMI<24 kg/m^2**	**BMI≥24 kg/m^2**
Effect	Mediation proportion, % (95%CI)	Mediation proportion, % (95%CI)	Mediation proportion, % (95%CI)
SBP	41.8 (37.2, 46.5)	-63.2 (-89.1, -37.2)	48.9 (39.0, 58.8)
RPG	21.7 (15.0, 24.6)	-9.48 (-14.0, -4.96)	11.0 (8.31, 13.7)
Direct Effect	36.5 (29.5, 43.5)	173 (143, 202)	40.1 (28.3, 51.9)

Proportions of path-specific effects were estimated using g-computation algorithm, including age, sex, region, education status, household income, current smoking, and alcohol drinking as covariates. Interaction effect by both mediators was no significant in all models, thus not presented. SBP=systolic blood pressure. RPG=random plasma glucose.

Table S8. Mediation analysis of delay time for all-cause mortality per SD increase of WHR/BMI in CKB

	WHR		BMI<24 kg/m^2		BMI≥24 kg/m^2	
	Estimation (95%CI)	Mediation proportion, % (95%CI)	Estimation (95%CI)	Mediation proportion, % (95%CI)	Estimation (95%CI)	Mediation proportion, % (95%CI)
**Model 1-2: Mediated by blood pressure (hypertension status)**						
TE	-1.62 (-1.88, -1.36)	28.8 (25.0, 34.2)	1.28 (0.96, 1.68)	-22.7 (-30.8, -16.7)	-1.39 (-1.78, -1.01)	36.6 (28.8, 51.4)
NDE	-1.15 (-1.40, -0.89)		1.57 (1.24, 1.96)		-0.88 (-1.26, -0.48)	
NIE	-0.47 (-0.51, -0.43)		-0.29 (-0.32, -0.26)		-0.51 (-0.58, -0.44)	
**Model 2-2: Mediated by blood glucose (diabetes status)**						
TE	-1.34 (-1.60, -1.08)	22.0 (18.6, 27.1)	1.26 (0.93, 1.65)	-6.41 (-9.02, -4.57)	-1.19 (-1.57, -0.80)	10.3 (7.48, 15.4)
NDE	-1.05 (-1.30, -0.78)		1.34 (1.00, 1.73)		-1.07 (-1.44, -0.68)	
NIE	-0.29 (-0.31, -0.27)		-0.08 (-0.10, -0.07)		-0.12 (-0.14, -0.10)	

Linear model was used for estimate exposure-mediator effect and accelerated failure time model was used to estimate exposure-outcome and mediator-outcome effects. The total effect represented the association between WHR/BMI on all-cause mortality. Natural indirect effect represented association mediated by the mediators (blood pressure or glucose). Natural direct effect represented association not mediated by mediators. Mediation proportion was calculated by NIE/TE. Effects were estimated by delay time for all-cause mortality. 95% confidence interval (95%CI) was estimated by quasi-Bayesian Monte Carlo simulation with 100 replications. All analyses were adjusted for age, sex, race, education status, household income,

current smoking, and alcohol drinking. TE=Total Effect. NDE=Natural direct effect. NIE=Naturel indirect effect. SBP=systolic blood pressure.

Table S9. Mediation analysis of delay time for all-cause mortality per SD increase of WHR/BMI in US NHANES

	**WHR**		**BMI<25 kg/m^2**		**BMI≥25 kg/m^2**	
	Estimation (95%CI)	Mediation proportion, % (95%CI)	Estimation (95%CI)	Mediation proportion, % (95%CI)	Estimation (95%CI)	Mediation proportion, % (95%CI)
**Model 1-2: Mediated by blood pressure (hypertension status)**						
TE	-5.97 (-8.24, -3.41)	9.67 (3.51, 18.7)	8.00 (4.32, 11.8)	-1.63 (-6.16, 1.38)	-3.94 (-6.32, -1.14)	10.7 (-0.27, 27.1)
NDE	-5.37 (-7.66, -2.94)		8.01 (4.36, 12.0)		-3.53 (-5.78, -0.95)	
NIE	-0.60 (-1.13, -0.18)		-0.14 (-0.45, 0.10)		-0.41 (-0.84, -0.01)	
**Model 2-2: Mediated by blood glucose (diabetes status)**						
TE	-5.32 (-7.55, -2.80)	13.0 (5.84, 23.0)	8.42 (4.75, 12.5)	-0.90 (-3.72, 1.07)	-3.28 (-5.50, -0.89)	13.8 (5.27, 59.0)
NDE	-4.63 (-7.04, -2.09)		8.32 (4.65, 12.2)		-2.84 (-5.04, -0.37)	
NIE	-0.69 (-1.08, 0.23)		-0.11 (-0.36, 0.09)		-0.44 (-0.70, -0.21)	
**Model 2-3: Mediated by blood glucose (fasting glucose) (N=9720)**						
TE	-5.98 (-9.73, -2.27)	12.2 (4.15, 31.0)	6.55 (2.12, 11.3)	-2.89 (-10.8, 0.90)	-1.90 (-5.91, 2.31)	23.0 (-346, 310)
NDE	-5.23 (-8.91, -1.46)		6.73 (2.20, 11.5)		-1.29 (-5.27, 2.99)	

NIE	-0.75 (-1.20, -0.40)		-0.18 (-0.44, -0.04)		-0.61 (-1.05, -0.26)	

Linear model was used for estimate exposure-mediator effect and accelerated failure time model was used to estimate exposure-outcome and mediator-outcome effects. The total effect represented the association between WHR/BMI on all-cause mortality. Natural indirect effect represented association mediated by the mediators (blood pressure or glucose). Natural direct effect represented association not mediated by mediators. Mediation proportion was calculated by NIE/TE. Effects were estimated by delay time for all-cause mortality. 95% confidence interval (95%CI) was estimated by quasi-Bayesian Monte Carlo simulation with 100 replications. All analyses were adjusted for age, sex, race, education status, household income, current smoking, and alcohol drinking. TE=Total Effect. NDE=Natural direct effect. NIE=Naturel indirect effect.

Table S10. Mediation analysis by cut-off value of 25 of BMI in CKB

	**BMI<25 kg/m²**		**BMI≥25 kg/m²**	
	Estimation (95%CI)	Mediation proportion, % (95%CI)	Estimation (95%CI)	Mediation proportion, % (95%CI)
**Mediated by blood pressure (SBP)**				
TE	1.21 (0.92, 1.44)	-49.0 (-66.4, -39.8)	-1.63 (-2.07, -1.26)	41.3 (33.5, 52.4)
NDE	1.80 (1.56, 2.03)		-0.96 (-1.37, -0.61)	
NIE	-0.59 (-0.63, -0.55)		-0.67 (-0.74, -0.60)	
**Mediated by blood glucose (RPG)**				
TE	1.23 (0.96, 1.46)	-11.0 (-15.2, -9.1)	-1.40 (-1.83, -1.05)	13.7 (10.8, 18.6)
NDE	1.37 (1.11, 1.60)		-1.21 (-1.61, -0.85)	
NIE	-0.14 (-0.14, -0.13)		-0.19 (-0.22, -0.17)	

Mediation proportion was calculated by (Natural Indirect Effect)/ (Total Effect). 95% confidence interval (95%CI) was estimated by quasi-Bayesian Monte Carlo simulation with 100 replications. All analyses were adjusted for age, sex, region, education status, household income, current smoking, and alcohol drinking. TE=Total Effect. NDE=Natural direct effect. NIE=Naturel indirect effect. SBP=systolic blood pressure. RPG=random plasma glucose.

Table S11. Subgroup analysis between all-cause mortality and WHR/BMI in CKB

	WHR	BMI<24 kg/m^2	BMI≥24 kg/m^2
Effect	Mediation proportion, % (95%CI)	Mediation proportion, % (95%CI)	Mediation proportion, % (95%CI)
**Mediated by blood pressure (SBP)**			
**Age**			
30-65	42.8 (36.6, 49.3)	-36.4 (-51.5, -28.7)	54.5 (43.1, 71.5)
≥65	24.7 (19.6, 32.0)	-62.8 (-215, -39.1)	39.4 (26.2, 68.4)
**Sex**			
Male	44.2 (34.2, 55.9)	-38.6 (-62.2, -26.6)	53.8 (36.6, 87.5)
Female	32.8 (25.8, 39.8)	-44.7 (-61.3, -33.5)	46.1 (32.9, 66.5)
**Region**			
Rural	34.5 (28.1, 40.9)	-40.0 (-53.2, -30.8)	56.4 (38.8, 90.7)
Urban	44.7 (32.7, 59.8)	-42.8 (-80.6, -27.8)	41.2 (28.7, 58.3)
**Mediated by blood glucose (RPG)**			
**Age**			
30-65	33.1 (28.0, 40.4)	-6.11 (-9.43, -4.90)	14.5 (11.3, 20.3)
≥65	42.2 (34.0, 55.6)	-26.4 (-95.2, -16.1)	22.8 (14.7, 46.1)
**Sex**			
Male	32.7 (25.1, 43.8)	-6.18 (-8.87, -4.10)	17.5 (11.9, 31.8)
Female	44.7 (33.7, 59.8)	-10.8 (-18.7, -7.60)	16.4 (11.9, 25.4)
**Region**			
Rural	34.2 (27.2, 42.7)	-7.29 (-10.4, -5.16)	18.7 (12.6, 34.8)
Urban	42.0 (30.2, 61.2)	-10.4 (-21.4, -6.67)	14.9 (10.9, 22.5)

Mediation proportion was calculated by (Natural Indirect Effect)/ (Total Effect). 95% confidence interval (95%CI) was estimated by quasi-Bayesian Monte Carlo simulation with 100 replications. All analyses were adjusted for age, sex, region, education status, household income, current smoking, and alcohol drinking, if appropriated. SBP=systolic blood pressure. RPG=random plasma glucose.

Table S12. Subgroup analysis between all-cause mortality and WHR/BMI in NHANES

	WHR	BMI<25 kg/m^2	BMI≥25 kg/m^2
Effect	Mediation proportion, % (95%CI)	Mediation proportion, % (95%CI)	Mediation proportion, % (95%CI)
**Mediated by blood pressure (SBP)**			
**Age**			
30-65	7.12 (1.17, 16.8)	-2.08 (-11.9, 3.38)	14.4 (3.26, 43.0)
≥65	2.14 (-1.84, 13.7)	-0.23 (-6.06, 6.68)	0.03 (-13.0, 5.84)
**Sex**			
Male	10.5 (4.76, 21.8)	4.34 (-57.1, 13.8)	7.98 (2.25, 14.1)
Female	2.64 (-4.34, 13.0)	-0.73 (-3.47, 0.57)	0.74 (-143, 263)
**Race**			
Hispanic	0.62 (-7.32, 5.28)	0.17 (-21.0, 19.6)	-1.63 (-38.0, 99.5)
Non-Hispanic White/Black	5.53 (2.14, 11.4)	-2.66 (-7.42, -0.54)	7.03 (0.19, 19.8)
Other Race	-3.66 (-561, 660)	-0.56 (-44.6, 16.4)	2.31 (-179, 117)
**Mediated by blood glucose (HbA_{1c})**			
**Age**			
30-65	9.56 (4.02, 21.6)	-0.77 (-9.19, 2.27)	14.3 (7.31, 29.8)
≥65	21.6 (4.44, 59.7)	-2.32 (-17.5, 1.27)	19.1 (-139, 164)
**Sex**			
Male	9.41 (4.22, 22.2)	-0.30 (-20.6, 7.87)	8.5 (3.52, 17.3)
Female	9.97 (-1.07, 34.3)	-0.71 (-3.12, 0.81)	-19.9 (-224, 134)
**Race**			
Hispanic	0.75 (-9.55, 10.2)	0.25 (-5.11, 19.6)	-2.21 (-74.2, 71.2)
Non-Hispanic White/Black	13.2 (6.23, 33.0)	-1.38 (-4.97, 0.24)	16.1 (8.6, 37.2)
Other Race	-2.46 (-104, 56.3)	-0.32 (-46.8, 26.1)	4.61 (-32.5, 73.7)

Mediation proportion was calculated by (Natural Indirect Effect)/ (Total Effect). 95% confidence interval (95%CI) was estimated by quasi-Bayesian Monte Carlo

simulation with 100 replications. All analyses were adjusted for age, sex, race, education status, household income, current smoking, and alcohol drinking, if appropriated. SBP=systolic blood pressure. HbA1c=glycated hemoglobin.

Table S13. Univariate mediation analysis of risk of all-cause mortality per SD increase of WHR/BMI in NHANES

	**WHR**	**BMI<25 kg/m^2**	**BMI≥25 kg/m^2**
Effect	Mediation proportion, % (95%CI)	Mediation proportion, % (95%CI)	Mediation proportion, % (95%CI)
Total cholesterol	-7.40 (-19.6, -2.11)	8.64 (3.59, 17.7)	1.43 (-0.95, 8.02)
Triglyceride	1.46 (-23.0, 20.8)	-0.63 (-16.9, 8.28)	8.41 (-14.9, 28.6)
Low-density lipoprotein cholesterol	-11.9 (-83.7, -3.44)	9.25 (-0.27, 27.6)	-1.11 (-125, 93.0)
High-density lipoprotein cholesterol	3.80 (-16.3, 18.5)	-5.30 (-14.2, 4.41)	8.41 (-14.9, 28.6)
C-reactive protein	6.48 (3.05, 11.9)	-0.93 (-3.22, -0.04)	10.85 (3.99, 33.8)
Uric acid	2.61 (-7.41, 13.3)	-6.93 (-19.9, 0.95)	4.1 (-9.35, 30.6)
Blood nitrogen	-1.26 (-9.22, 3.21)	-0.32 (-4.15, 1.62)	0.21 (-0.86, 3.07)
Serum creatinine	-6.54 (-16.0, -2.3)	-2.38 (-6.63, -0.25)	-1.25 (-6.13, 0.76)
Homocysteine	-2.14 (-6.53, 0.53)	1.19 (-3.83, 8.62)	0.48 (-2.76, 4.95)
Vitamin B12	-2.23 (-9.14, 2.01)	-0.67 (-7.65, 0.88)	-4.73 (-12.4, -0.93)
Total protein	-2.34 (-7.28, 1.49)	-0.08 (-5.68, 3.44)	-3.76 (-11.7, -0.33)
Albumin	27.2 (20.0, 41.0)	-9.48 (-34.0, -2.82)	27.0 (10.1, 74.7)
Alanine transaminase	4.45 (-6.97, 13.4)	-4.69 (-15.3, 1.02)	4.77 (-7.3, 23.5)
Aspartate aminotransferase	3.74 (1.58, 7.31)	-0.11 (-1.99, 1.02)	2.20 (0.02, 10.9)
White blood count	13.0 (1.06, 30.3)	-3.52 (-12.0, -0.15)	14.0 (2.14, 29.5)
Red blood count	-8.42 (-17.9, -5.09)	3.88 (-0.02, 11.9)	-14.4 (-53.6, -6.37)
Hemoglobin	-0.43 (-1.86, 0.74)	1.40 (-0.58, 4.98)	-0.04 (-2.09, 1.27)
Vitamin D	17.5 (9.96, 45.3)	-0.69 (-6.00, 1.92)	36.0 (-139, 208)

Mediation proportion was calculated by (Natural Indirect Effect)/ (Total Effect). 95% confidence interval (95%CI) was estimated by quasi-Bayesian Monte Carlo

simulation with 100 replications. All analyses were adjusted for age, sex, race, education status, household income, smoking, and alcohol drinking.
